# Validation of a flow cytometry based chemokine internalization assay for use in evaluating the pharmacodynamic response to a receptor antagonist

**DOI:** 10.1186/1479-5876-6-76

**Published:** 2008-12-01

**Authors:** Timothy Wyant, Alan Lackey, Marie Green

**Affiliations:** 1 Millennium Pharmaceuticals, Cambridge, MA, USA; 2 Esoterix Center for Clinical Trials, Brentwood, TN, USA; 3 Nodality Inc. Brentwood, TN, USA

## Abstract

Pharmacodynamic assays are important in clinical trial design to investigate the relationship between drug concentration (pharmacokinetics) and drug "effect' or biological activity. Increasingly flow cytometry is being used to examine the pharmacodynamic effect of new drug entities. However, to date, the analytical validation of cytometry based assays is limited and there is no suitable guidance for method validation of flow cytometry-based pharmacodynamic assays. Here we report the validation of a flow cytometry-based chemokine internalization assay for use in evaluating the effect of a receptor antagonist in clinical trials. The assay method was validated by examining the stability of the reagent, assay robustness, sensitivity, repeatability and reproducibility precision. Experimental results show the assay reagent was stable over 26 weeks. The assay demonstrated a sensitivity to distinguish 0.005 μg/ml of a CCR2 antagonist with a %CV of 13.3%. The intra-assay repeatability was less than 15% with an inter-assay repeatability of less than 20%. In vivo study results demonstrated that the assay was consistent and a reliable measure of antagonist activity.

## Background

Chemokines are a class of small proteins that have potent chemotactic activity for cells of the immune system. In addition, they have the ability to activate leukocytes, to stimulate cytokine and proteolytic enzyme production, to mediate angiogenesis, and may be involved in cell proliferation and death. [1] The chemokine receptor CCR2 is widely expressed on mononuclear cells and a subset of memory (CD45RO+) CD4+ helper T cells. Activation of CCR2 by monocyte chemoattractant protein-1 (MCP-1), the major CCR2 ligand, is known to mediate chemotaxis and degranulation of monocytes as well as migration of activated effector memory T cells. [2,3] The MCP-1/CCR2 pathway has been implicated in a variety of disease states such as Rheumatoid Arthritis, Multiple Sclerosis, and Atherosclerosis making the development of antagonists of this pathway an attractive pharmacological target [4-8]. Currently several companies have begun clinical trials of CCR2 antagonists [9].

*In vitro *pharmacodynamic assays are increasingly being utilized to demonstrate that a compound is having a desired biological effect after *in vivo *dosing. For CCR2 antagonists, the monitored effect is inhibition of either receptor signaling or ligand binding, depending on the mode of action of the drug being examined. When bound to their receptors chemokines, such as MCP-1, induce the receptor to internalize [2,3]. We have capitalized on this and developed a flow cytometry assay to measure MCP-1 ligand internalization in clinical trials as a measure of the pharmacodynamic effect of our CCR2 antagonist.

Unlike pharmacokinetic and immunogenicity assays [10-15], there has not been any regulatory guidance published on the essential parameters for validation of pharmacodynamic assays such as those based on flow cytometry. In the past, variations in instruments, instrument settings, reagents and population heterogeneity had made validating assays based on flow cytometry difficult. Fortunately, advances in instrument standardization protocols based on fluorescent beads, more user friendly instruments and a greater reagent and instrument control by manufacturers has now made it possible to address the criteria and rigor that would accompany a validated flow cytometry assay [16]. Using the guidance for ligand binding assays [12] as a foundation in which to base the validation of a flow cytometry pharmacodynamic assay and applying the "appropriate" parameters for a cell based cytometry assay, we validated a MCP-1 internalization assay. The parameters we examined included the stability of the reagents, the robustness, sensitivity, repeatability, precision and reproducibility of the assay. The precision was determined both in the *in vitro *validation phase and through retrospective analysis of in-study data.

## Methods

### Generation of Alexa 488 labeled MCP-1

Recombinant carrier free human MCP-1 was purchased from R&D Systems and fluorescently labeled with Alexa 488 (In Vitrogen Molecular Probes) using the conditions recommended for small proteins by Molecular Probes procedure. Alexa Fluor 488 was chosen due to the dye's increased stability and resistance to pH changes over a wide range of pH values (InVitrogen). The Alexa-488 labeled MCP-1 (AF488-MCP-1) was purified from the excess labeling reagent and free MCP-1 by RP-HPLC using a Vydac C18 semi-prep column (10 × 250 mm) and BioCad Vision Workstation. Labeled Peaks were identified and examined for their ability to bind to receptor positive cells. Identified peaks were pooled and retested for binding in a flow cytometry binding assay. The reagent was aliquoted, tested for freeze-thaw stability and frozen at -70°C.

### MCP-1 alexa 488 internalization assay

**Figure 1:**
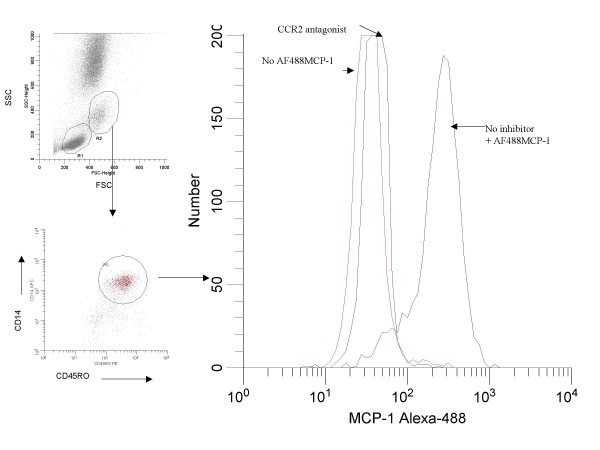
Representative histogram of AF488-MCP-1 staining in human whole blood. Cells were stained with AF488-MCP-1 in the presence (black) or absence of a CCR2 receptor antagonist (blue) and without the AF488-MCP-1 (red). Gating was based on the monocyte profile in forward and side scatter followed by gating on the CD14+ monocyte population.

Briefly, whole blood was incubated with AF488-MCP-1 for one hour at 37°C. Erythrocytes were lysed using PharmLyse (BectonDickenson) and the remaining white blood cells were briefly exposed to an acid salt wash (0.5 M NaCl, 0.2 M Acetic Acid, 0.5% sodium azide) by suspending the cells in 1 mL of solution for 5 minutes. This procedure was done to strip surface AF488-MCP-1 allowing only internalized AF488 MCP-1 to be observed. Samples were subsequently washed with PBS (pH 7.4) and a cocktail of anti-CD14 APC, anti-CD45RO PE, anti-CD4 PerCP was added to identify the CCR2 expressing monocytes and memory T cells during acquisition and analysis. Formaldehyde (1.5%) was added to fix the samples which were then analyzed on a flow cytometer (BD FACS Calibur). In one reaction, excess unlabeled MCP-1 was added prior to the addition of AF488-MCP-1 as a control. An example of the staining is in Figure 1. For most purposes the internalization assay was performed within 2 hours of blood draw. However, as part of the validation the ability to process the blood after 24 hours was examined (see below).

For the purpose of assay validation, whole blood collected from normal healthy volunteers was incubated *ex-vivo *either with or without the CCR2 antagonist prior to the addition of the fluorescent staining reagents. MESF (Mean Equivalence of Soluble Fluorescence) values were determined by utilizing standardized MESF calibration beads (Bangs Laboratories Fishers, IN).

### Assay validation

#### Overall criteria for evaluation

In general, for determination of %CV relevance, the guidelines established for ligand binding ELISA pharmacokinetic assays [10] was used to establish the %CV boundaries. A %CV less than 20% was considered an acceptable parameter. A 25% CV was used for values falling on the lower ends of curves. It was expected that fractional values such as that observed after saturation inhibition to have greater variability. Similarly, the inter-person variability was also anticipated to be greater and was to be documented here by retrospective analysis of phase one data.

#### Instrument set-up, MESF calibration and data analysis

A Becton Dickenson FACSCalibur instrument using 488 argon and red-diode lasers was calibrated daily using QC3 calibration beads (Bangs Laboratories). MESF was determined using the Quantum 1000 series bead sets from Bangs Laboratories daily. All raw instrument data was analyzed using WinList 5.0 (Verty Software House). Curve fitting and determination of EC_50 _and IC_50 _values was performed using Prism 4.0 (Graphpad) when applicable. The mean, standard deviation, standard error and % coefficient of variation (%CV) were calculated using Excel 2003 (Microsoft).

#### Reagent titration on whole blood

In order to determine the optimum reagent AF488-MCP-1 concentrations to use in the assay, a titration curve was performed. Serial dilutions of AF488-MCP-1 was added to 80 μL of whole blood and allowed to incubate at 37°C for 1 hour. The maximum internalization at 1 hour was determined to be the point at which no additional fluorescence was observed with increasing concentrations of AF488-MCP-1. For purposes of the *in vitro *validation, titration curves were generated by serially diluting a CCR2 antagonist into whole blood and incubating at room temperature for one hour prior to addition of the AF488-MCP-1. The CCR2 antagonist used here was an in house anti-CCR2 antibody which had been demonstrated to inhibit the binding and activity of MCP-1 in vitro (data not shown).

#### Stability

AF488-MCP-1 reagent stability was determined by examining both the binding of AF488-MCP-1 in whole blood over time and after five freeze-thaw cycles of the reagents. Stability of the AF488-MCP-1 was measured over a 26 week period at -70°C. Stock reagent stored at -70°C was diluted down to 150 nM, 100 nM, and 50 nM and added to whole blood (final concentration of AF488-MCP: 15 nM, 10 nM, 5 nM). Four different healthy volunteer blood donors were tested in the internalization assay at each time point and the resulting MESF and % positive values from each individual were averaged. Freeze-thaw (-70°C) stability was assessed by aliquoting the AF488-MCP-1 and cycling the various aliquots through different numbers of freeze-thaws. The cycled AF488-MCP-1 was subsequently utilized in the internalization assay and the resultant values for each cycle compared. Since the material was frozen after production the 1^st ^freeze thaw cycle is the baseline value from which all other freeze thaw values were compared.

#### Robustness and sensitivity

Assay robustness was defined as how "reproducibly" the assay performed over time within the same blood sample, or in other words, how well the assay can withstand deliberate manipulation of environmental influences. Since the whole blood samples were to be shipped to a processing site, robustness was addressed by assaying the internalization of bound AF488-MCP-1 in CD14 (+) and CD4+CDR45RO+ cells over time at 1 hour, 24 hours, 48 hours and 72 hours after *in vitro *spiking of whole blood samples. Changes in overall fluorescence or the percentage of cells able to internalize MCP-1 were compared to the one hour "fresh" sample. Additionally, in order to examine the effect of overnight shipping on inhibition of internalization of AF488-MCP-1 by the receptor antagonist a direct comparison of the effect of overnight storage on the IC_50 _of the CCR2 inhibitor was examined. Briefly, receptor antagonist was incubated with whole blood at ambient temperature for 24 hours followed by processing through the internalization assay. Results obtained from the overnight incubation were compared to results obtained by processing the whole blood after only one hour incubation with the CCR2 antagonist.

The sensitivity of the assay, or the ability of the assay to demonstrate inhibition of ligand internalization at low concentrations of CCR2 inhibitor, was determined by serially diluting the CCR2 antagonist into whole blood and incubating for 1 hour at room temperature. The curves generated from the results of a minimum of 4 individuals were averaged.

#### Precision (repeatability/reproducibility)

Assay reproducibility was determined by assaying internalization of AF488-MCP-1 from the 10 different donors' blood drawn at 3 different times (each individual drawn 3 times). The blood draws were spaced 2–4 days apart to allow for recovery of the donor prior to the next blood draw. Measuring the intra-individual donor repeatability was accomplished by performing the internalization assay in triplicate. The mean, standard deviation and % CV were calculated from triplicate values (intra-sample repeatability), for each individual over time (intra-person reproducibility/inter-assay repeatability), and across individuals (inter-person reproducibility).

#### In-study validation

108 individuals (54 placebos, 54 CCR2-antagonist treated) were assayed in the internalization assay over a 113 day in the absence (placebos) or presence (treated) of AF488-MCP-1 the CCR2 receptor antagonist. Volunteers were dosed with a single dose of either antagonist or vehicle control and whole blood was drawn, shipped overnight to the processing laboratory and assayed. Blood samples were drawn prior to dosing (pre) and immediately (within 5 minutes) following completion of the infusion on Day 1, and again on day 3 (9 individuals only), 8, 15, 29, 43, 57, 71, 85, and 113. All 108 (54 placebo and 54 dosed) individuals were assessed at all time points except day 3. Mean, standard deviation % CV and standard error for the data grouped across all placebos and placebos + pre-dose of all 108 individuals were examined. The pharmacodynamic effect was examined by plotting the internalization of AF488-MCP-1 in CD14+ monocytes and memory helper T cells (CD4+CD45RO+) after dosing with the CCR2 antagonist on the first day. The pharmacodynamic effect in the dosed group was measured throughout the period however; the pharmacokinetic/pharmacodynamic relationship is beyond the scope of this manuscript.

## Results

### Reagent titration

**Figure 2:**
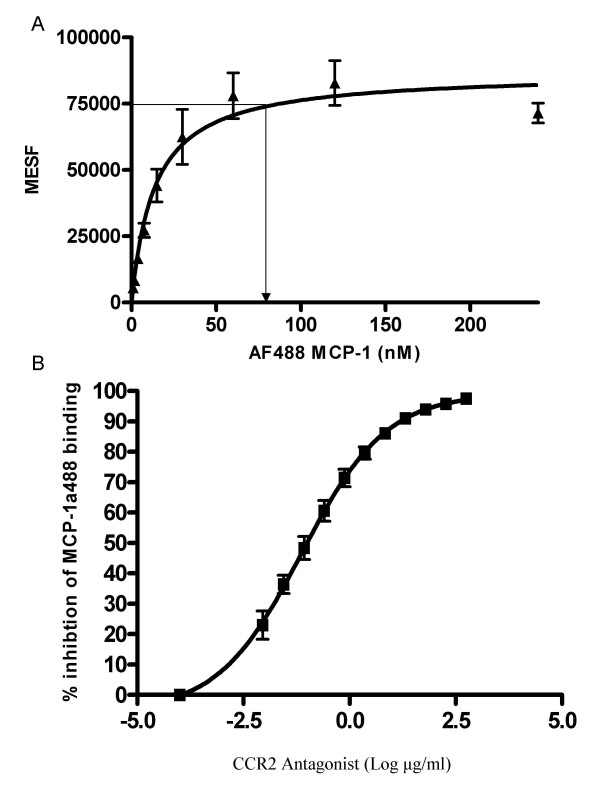
Titration of assay reagents. A) AF488-MCP-1 was serially diluted in whole blood and allowed to react at room temperature. CD14+ monocytes were examined and the Mean Equivalence of soluble fluorescence (MESF) was reported. Maximum saturation was determined to be 60–75 nM. B) Titration of CCR2 antagonist against optimum concentration (60 nM) of AF488 MCP-1

In order to determine the optimum concentration of AF488-MCP-1 to use in the assay the reagent was titrated on whole blood from 3 healthy volunteers and a titration curve was produced. As shown in Figure 2a, saturation of binding was achieved at a concentration of 60–70 nM of AF488-MCP-1. Since the internalization assay is to be used as a measure of pharmacodynamic effect of a CCR2 antagonist, it was also important to demonstrate the ability of the CCR2 antagonist to inhibit the saturating concentration of the AF488-MCP-1 used in the assay. To accomplish this CCR2 antagonist was titrated into the assay using the derived optimum AF488-MCP-1 concentration and an inhibition curve was generated. As shown in figure 2b, the CCR2 antagonist was able to inhibit the internalization of a saturating concentration of AF488-MCP-1. This result confirmed that 60 nM was the optimum concentration AF488-MCP-1 to use in the internalization assay.

### Reagent stability

**Figure 3:**
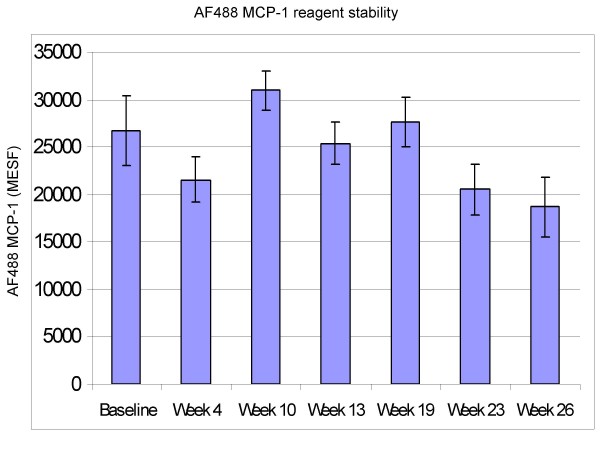
Stability of AF488-MCP-1: The ability of AF488-MCP-1 to bind and be internalized was examined over a 26 week period starting from 6 weeks post material production. Data represents the mean of 4 different individuals per time point. No significance was observed between baseline and week 4 (p = 0.15) or week 4 and week 26 (p = 0.34, paired 2 sided analysis).

The stability of the AF488-MCP-1 reagent, stored at -70°C, was determined in the whole blood internalization assay by performing the assay on 4 different volunteers (differing at each time point) over a period ending at 26 weeks. The baseline value represents 6 weeks post manufacture of the reagent. The results demonstrate consistent staining despite prolonged storage of the AF488-MCP-1 at -70°C (Figure 3). There appeared to be a 20–30% drop in intensity of fluorescence (MESF) at the 26 week time point however, the overall results suggest this drop may be more of a reflection in donor variability rather than stability of the reagent (the same drop was observed at 10 weeks yet at 16 weeks the intensity was higher than that at 6 weeks). There was no significant difference between the MESF value obtained at baseline and week 4 (p = 0.15) or between week 4 and week 26 (p = 0.34).

In addition the AF488-MCP-1 was also assayed after five freeze/thaws. Using the same whole blood sample, there did not appear to be an effect of the first 4 freeze thaw cycles on internalization (as shown by a lack in the decrease in MESF or percent positive) (Table 1) as compared to initial freeze-thaw. However, an 11.8% decrease in MESF value was observed after the 5^th ^freeze thaw/cycle. Due to this 12% decrease at the 5^th ^freeze thaw cycle, it was decided that no greater than 4 freeze thaw cycles would be permitted with the material.

**Table 1:** Freeze thaw stability of AF488-MCP1

	MESF	Percent positive
Negative Control	2162.97	0.45

1 Freeze/Thaw	16844.06	98.59

2 Freeze/Thaw	16457.7	98.37

3 Freeze/Thaw	16249.23	98.63

4 Freeze/Thaw	17208.48	98.95

5 Freeze/Thaw	14850.99	97.8

### Assay robustness and sensitivity

**Figure 4:**
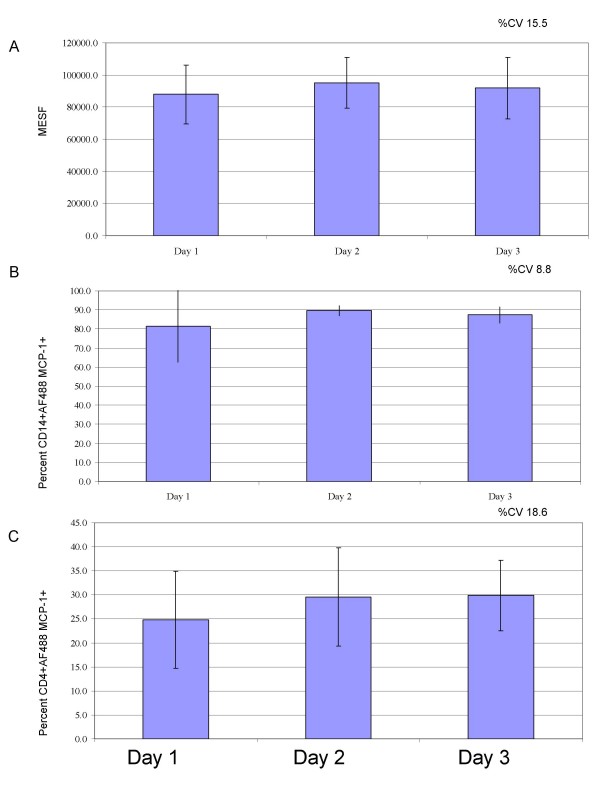
Robustness of assay over 3 days. The AF488-MCP-1 internalization assay was tested in whole blood over a 3 day period. CD14+ monocytes and CD4+ memory T cells were examined and the Mean Equivalence of Soluble Fluorescence (MESF) of the CD14+ cells (A) or percentage of CD14+ cells (B) or CD4+ (C) staining positive cells were examined. The percent CV of the parameters over the three day period (n = 5) are 15.5,8.8 and 18.6 respectively. Day 1 represents the initial baseline comparative value.

The robustness of the assay was examined using blood from 5 individuals at different time points: "fresh" (within 1 hour of blood draw), 24 hours, 48 hours and 72 hours post blood draw. This was performed both with and without the addition of the CCR2 antagonist. As shown in Figure 4, there was little change in the percentage of CD14+ cells staining positive for AF488-MCP-1 (80.8 ± 1.2%) or in the relative level of fluorescence (91612.3 ± 17543.1 MESF) observed over the 72 hour period. The variability across the time points was 15.8%. This variability is within that observed between individuals (16.3%–18.2%). A similar result was observed for the percentage of CD4+CD45RO+ cells staining positive for AF488-MCP-1 (17.6 ± 0.9%) (Figure 4).

It was determined that due to extremely low fluorescence values using MESF as an analytical measure on memory T cells (CD4+CD45RO+) cells was not reproducible (MESF range on CD4+CD45RO+ cells was 0 to 617 MESF with an average of 320 as compared to CD14+ MESF range of 53054 to 75851 averaging 61853 MESF). For this reason MESF values for the memory T cell population are not reported.

**Figure 5:**
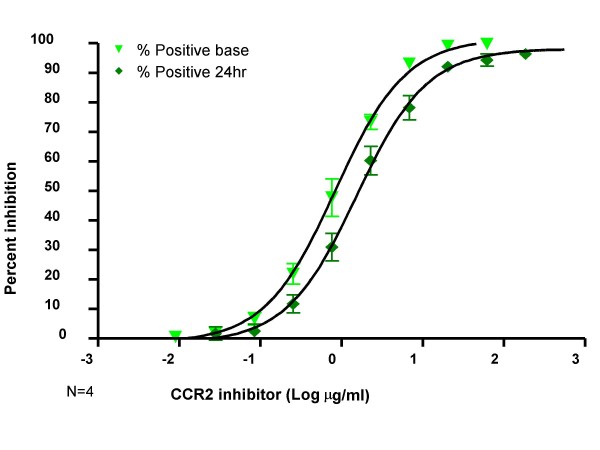
Robustness of assay: comparison of AF488-MCP-1 internalization inhibition at 1 hour and 24 hours. The AF488-MCP-1 internalization assay was tested in whole blood after one hour incubation with a serial dilution of CCR2 inhibitor and compared to incubation with the inhibitor for 24 hours. The IC50 for 1 hour was determined to be 0.9 ± 0.3 μg/ml compared to 1.6 ± 0.6 μg/ml (n = 4). Inhibition of percent positive cells shown with similar results observed for MESF.

Results from the experiments to investigate potential effects of shipping overnight or inhibition of AF488-MCP-1 internalization after 24 hours incubation with antagonist demonstrated that there was not a significant difference between the IC_50 _of inhibitor at 1 hour and 24 hours (Figure 5) (0.9 ± 0.3 μg/ml vs. 1.6 ± 0.6 μg/ml pvalue = 0.15).

Assay sensitivity, the ability to reliably demonstrate inhibition of internalization at low concentrations of inhibitor, was determined by serially diluting CCR2 into whole blood and determining the concentration at which the assay %CV of inhibition became greater than 20% and using the dilution below that point as the sensitivity. As shown in Table 2, at the lowest concentration of CCR2 inhibitor examined the % CV was 13.3% and therefore since the %CV was never greater than 20%, the sensitivity of the assay was determined to be the lowest titration point (0.005 μg/ml) of CCR2 antagonist.

**Table 2:** Percent Inhibition of monocyte internalization of AL488-MCP-1 with a CCR2 inhibitor

CCR2 inhibitior (μg/ml)	Vol A	Vol B	Vol C	Vol D	Average	Stdev	%CV
555.560	96.50	95.48	95.49	95.16	**95.66**	**0.58**	0.61

185.187	95.51	95.44	94.25	94.53	**94.93**	**0.64**	0.67

61.729	92.82	92.76	92.64	93.63	**92.96**	**0.45**	0.49

20.576	90.89	91.16	90.10	90.00	**90.54**	**0.58**	0.64

6.859	84.82	86.28	85.47	86.23	**85.70**	**0.69**	0.81

2.286	78.30	76.71	81.54	79.55	**79.03**	**2.04**	2.58

0.762	71.21	75.22	71.71	69.85	**72.00**	**2.29**	3.18

0.254	59.49	67.06	62.80	59.43	**62.20**	**3.61**	5.80

0.085	43.23	57.00	49.56	47.31	**49.28**	**5.78**	11.73

0.028	31.18	38.57	36.08	35.06	**35.22**	**3.07**	8.72

0.009	23.76	24.64	21.35	24.84	**23.65**	**1.60**	6.77

0.005	13.81	15.68	11.31	13.44	**13.56**	**1.79**	13.22

Based on MESF values

### Assay precision

The assay was performed using whole blood from 10 healthy volunteers which were drawn 3 times over a 2 week period. As shown in Table 3, CD14+ monocyte MESF values for AF488-MCP-1 ranged from 51476–115497 and were observed across all time points of the 10 individuals. Overall, 8 out of 10 individuals had %CVs of less than 20% with an average % CV of 15.5% across all 10 individuals. The percentage of CD4+CD45RO+ cells internalizing AF488-MCP-1 ranged from 11.7%–44.6%. Similar to the CD14+ monocytes, 8 out of the 10 individuals had %CVs of less than 20% with an overall average of 16.3% (Table 4). The CVs generated for replicate analysis (triplicate runs of all 10 individuals at all time points) are shown in Table 5. There was consistently a variability of less than 15% in all the assay parameters tested. In particular, the triplicate MESF data derived from the CD14+ cells had variability of less than 10% across all individuals and all days (Table 5).

**Table 3:** Reproducibility of Monocyte MESF

**volunter**	**day 1**	**day 2**	**day 3**	**average**	**Stdev**	**%CV**
**A**	61940	87026	91080	80015.4	16017.8	20

**B**	91392	122966	115497	109952	19147.8	17.4

**C**	51476	99899	103507	84960.7	25690.2	30.2

**D**	96517	90421	108916	98617.8	14227.2	14.4

**E**	78818	66928	68908	71551.6	8026.7	11.2

**F**	97349	89816	88213	91792.6	6327.6	6.9

**G**	103644	114153	114181	110659.2	8417.7	7.6

**H**	88285	96544	92983	92604	5061.8	5.5

**I**	103690	101193	74133	93005.3	16437.9	17.7

**J**	105782	82502	60611	82964.7	19887.1	24

* Day 1 refers to the initial baseline value obtained from freshly obtained whole blood.

All samples were processed within one hour of blood dra

**Table 4:** Reproducibility of Percentage of Memory T cells Positive

**volunter**	day 1*	**day 2**	**day 3**	**average**	**Stdev**	**%CV**
**A**	30.6	36.0	33.4	33.3	2.7	8.1

**B**	18.6	24.4	23.7	22.2	3.2	14.2

**C**	11.7	44.6	33.8	30.0	16.8	55.8

**D**	29.8	23.6	29.1	27.5	3.4	12.3

**E**	36.4	30.4	38.2	35.0	4.1	11.7

**F**	13.5	15.4	18.1	15.7	2.3	14.8

**G**	27.3	29.6	28.3	28.4	1.2	4.1

**H**	40.9	41.9	39.8	40.9	1.1	2.6

**I**	13.6	14.3	20.4	16.1	3.7	23.2

**J**	25.5	35.0	34.0	31.5	5.2	16.6

* Day 1 refers to the initial baseline value obtained from freshly obtained whole blood.

All samples were processed within one hour of blood draw.

**Table 5:** Average % CV of triplicate test of 10 individuals over 3 days

	CD14+MCP-1al+		CD4+MCP-1al+	
	
	%	MESF	%	N/A
Day 1	5.6	8.9	11.9	N/A

Day 2	2.2	8	13.9	N/A

Day 3	3	7.4	7.7	N/A

### In-study results

**Figure 6:**
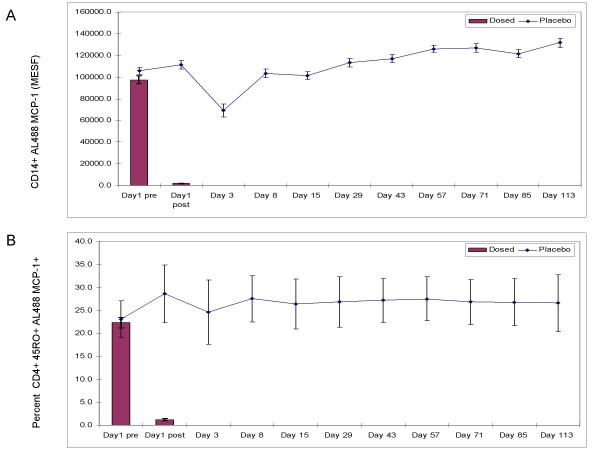
In study validation. AF488-MCP-1 internalization assay was performed on whole blood of individuals either dosed with a CCR2 antagonist or placebo. Various time points from pre-dose through 113 days were examined for the ability of the assay to distinguish antagonist response. Shown here are the CD14+ monocytes MESF (A) and percentage of CD4+CD45RO+Memory T cells (B) staining for AF488 MCP-1 internalization. Reported values are the mean ± SE of the 54 individuals per group with the exception of day 3 were the n = 9. Bar graphs demonstrate the overall drug effect on the assay post dose on day one. The longitudinal pharmacodynamic effect is beyond the scope of this paper and therefore subsequent values are not shown.

This assay was used as a pharmacodynamic marker for biological activity of a CCR2 antagonist in a clinical trial consisting of 108 healthy individuals. The data generated from this study was used retrospectively to further validate the internalization assay. Fifty four individuals were given a single dose of a CCR2 antagonist and 54 individuals were given a vehicle control placebo. Blood was drawn at various time points throughout 113 days and the ability of monocytes and memory T cells to internalize AF488-MCP-1 was measured and examined for reproducibility over time, across individuals (population heterogeneity) as well as the ability of the assay to demonstrate a pharmacodynamic effect. As shown in figures 6a and 6b (bar graphs), shortly after dosing on day 1 there was a complete and rapid inhibition of internalization in both CD14+ monocytes and CD4+CD45RO+ memory T cells from the group receiving the CCR2 antagonist. In contrast, there was no inhibition of internalization in the placebo (Figures 6a and 6b placebo line). An effect was observed at day 3 (up to 40% decrease in internalization of AF488-MCP-1) however, only 9 of the 54 individuals were sampled at the day 3 time point leading to a potential sampling bias in the data. At all other time points, all 108 (54 placebo and 54 dosed) individuals where assessed using the internalization assay. The assay variability in the placebo data is 15.5% over the 113 days of sampling (day-3 time point included for a total of 528 samples) (Table 6). This data suggests that over a 113 day period there is relatively little change in the overall expression of the CCR2 receptor as well as the cell's capacity to internalize the ligand.

**Table 6:** In study intra and inter-person variability

All Pre-dose samples					
	average	Stdev	%CV	n	Sterror
	
					
Monocyte MESF	101586.6	38366.8	37.8	108	3691.8

Percentage of Memory T cell	22.7	0.7	3.2	108	0.3

All Placebo over 113 days					
	
	average	Stdev	%CV	n	Sterror

Monocyte MESF	111589.34	17265.61	15.47	528	4856.303

The inter-person reproducibility of the assay was demonstrated by testing the pre-dose samples of all 108 individuals. The average fluorescence MESF value for monocytes was 101586.5 ± 38366.8 across all pre-dose samples (Table 6). The observed variation in the fluorescence values for the CD14+ cells across the population was 37.8%. The percentage of CD4+CD45RO+ T cell staining positive for AF488 MCP-1 internalization was 22.7 ± 0.7 giving a 3.2%CV for all of the individual pre-dose values.

## Discussion

The ability to demonstrate biological activity of drug (pharmacodynamic biomarkers) has become a valuable measurement in the development cycle of a pharmaceutical. For example, changes in lipid profiles, CRP and blood pressure have been used as pharmacodynamic (PD) measures for the assessment of drug treatment effect [17-21]. Pharmacodynamic assays such as the one described here are important for the overall clinical development of a pharmaceutical entity for which target effects are not easily identified *in vivo*. The ability to confidently and reliably demonstrate the action of drug on target enables the researcher early on to evaluate drug effects. Unfortunately, many of the mechanistic pharmacological effects have been difficult to observe *in vivo *and/or have been limited by the availability of reliable techniques for measuring the effect.

Flow cytometry has been a proven useful tool in the diagnosis of hematological disorders [22] as well as the diagnosis and monitoring of progression for diseases such as AIDS [23] and hematological cancers [24]. Recent advances in instrument platforms, reagent quality and the increase in clinical usage have driven flow cytometry to be highly reproducible and consistent. For these reasons, flow cytometry has become an excellent platform for pharmacodynamic assays.

Cytometry can measure both phenotypic and functional parameters from whole blood. To date, the use of flow cytometry in clinical trials has for the most part been limited to measuring changes in phenotypic profiles and cell populations in response to therapeutics. However, several examples of flow cytometry assays being used to measure PD effects have recently been published. Ebo et al have recently published the validation of a flow cytometry assay showing the antagonistic effect of a compound on the ability of basophils to undergo a shape change [25]. Similarly, Kelly et al has demonstrated the effect of an anti-CD40 antibody on the levels of circulating B cells in cynomolgus monkey [26]. While the examples noted have demonstrated the power and versatility of flow cytometry in clinical and pre-clinical trials, to date, there has been no guidance or white papers released addressing validation of pharmacodynamic assays, particularly assays based on flow cytometry. This is in contrast to other assays used in drug development such as ligand binding and immunogenicity assays [10-15]. Additionally, there has been a recent white paper published in which recommendations were made on the use and validation of conventional biomarker assays in clinical trials [27,28]. To date, no parameters for validating specifically flow cytometry PD assay have been proposed.

These guidance and white papers can serve as a template or guide for the validation of a flow cytometry PD assay. Flow cytometry PD assays should also demonstrate similar parameters: reagent stability (to ensure reagents remain consistent from day to day), assay robustness (how the assay is affected by variables such as overnight shipping), sensitivity (how the assay responds to low concentrations of compound i.e. the drug effect threshold), repeatability (the variability of results when the assay is performed multiple times on the same sample (inter-assay) and in replicate (intra-assay)) and reproducibility precision (the variability of results from blood drawn from the same individual multiple times and also from different individuals). Accuracy, as classically defined for ELISA and mass spectrometry based technologies, is the ability of an assay to measure analyte against a standard or quantitative control (QC) and is the most difficult of the parameters to define for flow cytometry based assay because the controls that would allow one to address this parameter directly (such as fixed cells of known antigen expression and density) do not exist or are untried for this purpose. However, several bead based methods by which instrument precision can be measured do exist. These bead based procedures not only standardize instrument settings accounting for daily fluctuations but also include fluorescence intensity standards to which to relate the results allowing greater reproducibility of data from instruments across laboratories and over time [16]. The lack of standard controls for each parameter examined in flow cytometry limits the ability of the assay to be used in a quantitative manner and at best renders it semi-quantitative. For this reason, analysis in longitudinal clinical trials may best be served by examining the value observed relative post exposure to a baseline pre-drug treatment value. It is therefore critical that the variability of the cytometric assay be well understood prior to initiation of a clinical trial. Further refinement of the longitudinal variability during phase 1 trials solidifies our confidence in the assay and allows the scientist to better define the limits of what observed effects can be considered as a direct result of the therapy and not due to variations in the assay.

The purpose of validating an assay is to be able to demonstrate that the assay not only scientifically addresses the questions (Fit-for-Purpose [27]) but also does so in a reproducible and reliable manner. To this end, a whole blood alexa-488 labeled MCP-1 internalization assay was validated here for use in clinical trials investigating a CCR2 antagonist by examining a standard reagent concentration to use, the stability of the reagent, the robustness of the assay, the reproducibility across individuals over time, and the intra-assay repeatability through replicate analysis. Since this is a whole blood assay other parameters such as matrix effects were not examined However, parameters such as these are important to consider when moving from differing disease states and may require cross validations in new disease state whole blood matrix [12,27].

The AF488-MCP-1 internalization assay described here was shown to be sensitive, robust, repeatable and reproducible. The assay is able to demonstrate a pharmacodynamic effect after *in vivo *dosing and additionally established that expression of CCR2 on both monocytes and memory T cells is relatively stable over 113 days using a controlled flow cytometry platform. The AF488-MCP-1 internalization assay will be an important tool which allows the clinical researcher to determine CCR2 saturation and a pharmacodynamic-pharmacokinetic relationship in clinical trials investigating CCR2 ligand antagonists.

## Competing interests

Drs. Wyant and Green are employees of Millennium Pharmaceuticals at which the research was completed. Mr. Lackey formally worked at Esoterix and is now at Nodality. The authors list no competing interest.

## Authors' contributions

TW designed and carried out experiments and drafted the manuscript. AL carried out the assay in the clinical trials. MG aided in the design of the experiments and review of the manuscript. All authors read and approved the final manuscript.
